# Gaza Ceasefire: Improve WASH, Promote Cooperation

**DOI:** 10.3389/ijph.2024.1607412

**Published:** 2024-05-01

**Authors:** Oliver Razum, Hazem Agha, Nadav Davidovitch, Timothy McCall, Stav Shapira

**Affiliations:** ^1^ School of Public Health, Bielefeld University, Bielefeld, Germany; ^2^ Faculty of Public Health, Al-Quds University, Jerusalem, Palestine; ^3^ The Association of Schools of Public Health in the European Region (ASPHER), Brussels, Belgium; ^4^ Medical School OWL, Bielefeld University, Bielefeld, Germany

**Keywords:** water and sanitation, Israel, Palestine, prevention, peacebuilding

“Water for prosperity and peace” is the title of the United Nations World Water Development Report 2024 [1]. The motto is timely, and the association between water and peace works in two directions: adequate water supply is a prerequisite for peaceful and prosperous life. The present conflict in the Middle East shows that peace, or at least a ceasefire in Gaza, would also provide an urgently needed opportunity for improving water, sanitation, and hygiene (WASH) in the region. Such efforts would benefit Palestinians in Gaza, but also the Israeli population. Joint WASH-related efforts offer an opportunity to address public health needs and contribute to long-term cooperation, stability, and—in the long run—prosperity in the region.

Reports about civilians in Gaza tend to focus on deaths, injuries, destroyed residential areas, and imminent famine [2, 3]. Regional public health threats related to clean water and the sanitation crisis do not receive similar attention. Drinking water supply in both Israel and Gaza has heavily relied on desalination plants and groundwater wells; both require a reasonably clean environment to work in. In the past, the release of large quantities of untreated sewage from Gaza to the Mediterranean threatened the public health and water security of Palestinians and Israelis alike. Before 7 October, intensive investments made by the international community resulted in most of Gaza’s sewage being treated. Post 7 October, almost none of Gaza’s sewage treatment infrastructure operates, and raw sewage will ultimately again present a major public health concern for the populations of Gaza, Israel, and Egypt [4].

In Gaza, most desalination plants are damaged or inoperable because of interrupted energy supply. An unusually wet winter that allowed extensive rainwater harvesting to complement water supply is ending. Table 1 shows the decline in water supply since 7 October in an area already experiencing chronic water scarcity [5]. With reductions by 80% or more, the decline is massive. All water sources are affected, either directly (such as the water pipes from Israel being interrupted) or indirectly due to lack of energy (e.g., because electricity supply from Israel is cut). In consequence, there is far too little water for drinking and washing—in some areas only 10% of the 15 Litres per person per day required in emergencies [3]. Most available water is of poor quality and frequently contaminated. This increases the risk of waterborne disease outbreaks [3], e.g., Hepatitis A and cholera, which may cross borders [4, 6, 7]. Calls for increasing water supply to Gaza and improving WASH could thus receive support from all parties involved as a shared interest.

**TABLE 1:** Gaza water supply, rounded estimates (data source: Palestine WASH cluster minutes of meeting, 13 March 2024, https://response.reliefweb.int/palestine/water-sanitation-and-hygiene accessed 11 April 2024; own calculations).

Source	Capacity unit^a^	Capacity		Capacity change (%), to 13 March 2024		Description	Challenge/opportunity
		Before 7 October 2023	13 March 2024	Loss per source	As part of subtotal loss^b^		
Israeli-supplied piped water	m^3^	52,800	10,000	−81%	−14%	1 of 3 water pipes partially functional	Reopen Israeli water supply pipes
	Litres/person/day	23	4				
Gaza desalination plants	m^3^	22,000	2,731	−87%	−6%	2 of 3 desalination plants partially operational	Repair desalination plants and provide energy to run them
	Litres/person/day	10	1				
Decentral wells	m^3^	300,000	51,741	−83%	−80%	Mostly municipal, large proportion with brackish water; others UNRWA and private	Provide energy to operate intact wells in areas still inhabitable; provide chlorine tablets, etc.
	Litres/person/day	133	23				
Subtotal	m^3^	374,800	64,472	−83%	—		
	Litres/person/day	161	28				
Newly installed desalination plant	m^3^	0	2,400^c^	—	<+1%	UAE desalination plant, Egyptian side of Gaza border	Massively increase emergency water provision places with population concentration (presently Southwest Gaza) also by sources other than new desalination plants
	Litres/person/day	0	1				
Total	m^3^	374,800	66,872	−82%	—		
	Litres/person/day	166	29				

^a^ Denominator population for *per capita* calculation: 2.26 million (data source: Palestine Ministry of Health, Health Annual Report, 2021 accessed 9 April 2024).

^b^ Subtotal capacity loss: 374,800 m^3^–64,472 m^3^ = 310,328 m^3^.

^c^ Equivalent to 15 Litres/person/day for 160,000 persons.

Several steps to improve WASH are technically feasible almost immediately [8], and by stressing the benefit to both Palestinians and Israelis could also be made politically feasible. Firstly, all three Israeli pipes supplying water to Gaza should be repaired and re-opened; five NGOs in Israel have appealed to Israel’s High Court for the re-opening in early April, and in the hearing, the state has declared its intention to do so (as of 21 April, this has not yet happened) [9]. Wells and small off-grid groundwater desalination facilities should be repaired; and water supply via the Egyptian side of Rafah needs to be massively increased by all means including additional desalination facilities (see Table 1). Second, sewage pumps should be repaired or replaced. In both cases, the politically contentious issue of energy supply needs to be tackled, possibly with temporary off-grid solutions. Third, more trucks with WASH equipment need to enter Gaza. A fourth mid-term undertaking is the emergency repair of sewage treatment plants and other WASH infrastructure. In the longer run, a shared economy for water is needed in the region, comprising three elements: “shared notions of water justice, normative economic practices for the exchange and distribution of water, and the associated social pressures that keep these norms in place.” [10].

Providing aid to civilians in Gaza has become a highly contentious matter in Israel because Hamas refuses to free Israeli hostages without a permanent ceasefire. Focusing on WASH offers a unique opportunity to improve living conditions of all parties, including the hostages. “Water … can promote peace,” says the UN report [1]. As a team of scientists from the region and beyond we conclude more carefully with the present conflict in view, but still hopefully: water and sanitation may indeed become a starting point for re-establishing constructive cooperation in the region, and ultimately a building block for promoting peace.

## References

[1] United Nations. The United Nations World Water Development Report 2024: Water for Prosperity and Peace. Paris: UNESCO (2024). Available from: https://reliefweb.int/report/world/united-nations-world-water-development-report-2024-water-prosperity-and-peace-enarruzhhiitdeko?gad_source=1&gclid=CjwKCAjwz42xBhB9EiwA48pT7ww0lW4UgUg3l1ebrURshR2loR-iPEDLotIGNl8Vk93QG1DdTk_GgBoC2voQAvD_BwE (Accessed April 19, 2024).

[2] World Food Programme (WFP). WFP Deputy Chief Warns Security Council of Imminent Famine in Northern Gaza Unless Conditions Change (2024). Available from: https://www.wfp.org/news/wfp-deputy-chief-warns-security-council-imminent-famine-northern-gaza-unless-conditions-change (Accessed April 19, 2024).

[3] United Nations Office for the Coordination of Humanitarian Affairs (OCHA). Hostilities in the Gaza Strip and Israel (2024). Available from: https://www.unocha.org/publications/report/occupied-palestinian-territory/hostilities-gaza-strip-and-israel-flash-update-94 (Accessed April 19, 2024).

[4] Efron S Fischbach JR Blum I Karimov RI Moore M. The Public Health Impacts of Gaza’s Water Crisis: Analysis and Policy Options. Rand Health Q (2019) 8(3):10. 10.7249/RR2515 31205810 PMC6557038

[5] EFE. Gaza Turns to Desalination amid Increasingly Scarce Water Access (2023). Available from: https://efe.com/en/other-news/2023-09-08/gaza-turns-to-desalination-amid-increasingly-scarce-water-access/ (Accessed April 19, 2024).

[6] Burki T. The Great Cholera Vaccine Shortage. The Lancet (2024) 403(10430):891–2. 10.1016/S0140-6736(24)00467-7 38461848

[7] Hermesh B Ma’ayan M Davidovitch N. Health Risks Assessment for the Israeli Population Following the Sanitary Crisis in Gaza. *Be’er Sheva: School of Public Health* . Israel: Ben-Gurion University (2019).

[8] The Israeli Institute for Regional Foreign Policies (Mitvim). Securing Critical Infrastructure in Gaza Is a Necessity for Israeli National Security (2023). Available from: https://mitvim.org.il/en/publication/securing-critical-infrastructure-in-gaza-is-a-necessity-for-israeli-national-security/ (Accessed April 19, 2024).

[9] Jewish News Syndicate (JNS). Israeli High Court Hears Petition Demanding More Aid to Gaza (2024). Available from: https://www.jns.org/israeli-high-court-hears-petition-demanding-more-aid-to-gaza/ (Accessed April 19, 2024).

[10] Beresford M Wutich A Garrick D Drew G. Moral Economies for Water: A Framework for Analyzing Norms of Justice, Economic Behavior, and Social Enforcement in the Contexts of Water Inequality. WIREs Water (2023) 10(2):e1627. 10.1002/wat2.1627

